# Instructor-led distance learning for training students in cardiopulmonary resuscitation: A randomized controlled study

**DOI:** 10.1371/journal.pone.0251277

**Published:** 2021-05-06

**Authors:** Sangsoo Han, Hye Ji Park, Sangun Nah, Eun Hae Lee, Hyun Ji Lee, Ju Ok Park, Choung Ah Lee

**Affiliations:** 1 Department of Emergency Medicine, Soonchunhyang University Bucheon Hospital, Bucheon, Republic of Korea; 2 Department of Emergency Medicine, Dongtan Sacred Heart Hospital, Hallym University, Hwaseong-si, Gyeonggi-do, Republic of Korea; 3 Division of Injury Prevention and Control, Korea Disease Control and Prevention Agency, Cheongju-si, Chungcheongbuk-do, Republic of Korea; St. Michael’s Hospital, CANADA

## Abstract

**Study hypothesis:**

Cardiopulmonary resuscitation (CPR) training can increase the likelihood of patient survival and better neurological outcomes. However, conventional learning (CL) has cost, time, and space constraints. This study aimed to evaluate whether laypersons who completed instructor-led distance learning (DL) acquired a level of CPR skill comparable to that achieved via CL training.

**Methods:**

This randomized controlled study recruited students from 28 Korean high schools who were randomized to complete instructor-led DL or CL training. The CL training involved classroom-based face-to-face training, whereas the instructor-led DL training was provided online using a videoconferencing system.

**Results:**

The study enrolled 62 students who were randomized to the CL group (31 participants) or the DL group (31 participants). Relative to the CL group, the DL group achieved remarkably similar results in terms of most CPR variables. In addition, the DL group had a significant improvement in the mean compression depth (before: 46 mm [interquartile range: 37–52 mm] vs. after: 49 mm [interquartile range: 46–54 mm], p<0.001).

**Conclusions:**

Instructor-led DL can be a suitable alternative to CL for providing CPR training to laypersons. In settings like the current COVID-19 pandemic, where face-to-face CL is not practical, DL may be a useful tool for delivering CPR training.

## Introduction

Cardiopulmonary resuscitation (CPR) performed by a bystander is one of the most important prognostic factors for out-of-hospital cardiac arrest (OHCA). CPR training improves bystander CPR rates, leading to a higher possibility of the return of spontaneous circulation (ROSC), more likelihood of survival, and better neurological outcomes [1,2]. Conventional CPR training is instructor-led and classroom-based, which creates challenges in terms of time, cost, logistics, and instructors’ and students’ discomfort being in a classroom environment [3]. Technological advances have created new vehicles for delivering training, and several studies have evaluated different CPR training methods, including distance learning (DL) [3–5]. In this context, DL is defined as the use of computer technology to provide training, which includes technical support that includes both online and offline learning [6]. The global COVID-19 pandemic has reduced opportunities to provide conventional training, which has increased interest in using DL to maintain medical education during the closure of educational institutions [7]. Thus, during the COVID-19 pandemic, the European Resuscitation Council has recommended discontinuing in-person basic life support (BLS) training for laypersons and has recommended DL-based CPR training to minimize the risk of infection and transmission [8]. However, there is limited research regarding whether DL can improve the quality of layperson CPR. Previous studies of DL for CPR training have used video-based and self-instruction methods, which provide online videos and allow the student to follow the course [9,10]. However, self-instruction methods lack instructor feedback, and it is possible that students might learn improper techniques (e.g., the wrong compression location).

This study aimed to evaluate instructor-led DL for CPR training and compare its results to those of conventional CPR training. This information may be useful for determining whether instructor-led DL is effective for improving CPR quality among students, which may translate into better outcomes after bystander CPR for OHCA.

## Materials and methods

### Study design and setting

This prospective, randomized, controlled study compared conventional and DL CPR training for laypersons using a manikin. To assign sample numbers equally to each group, block randomization was performed with the block size. The study was performed at a training center that administers CPR training courses that are approved by the Korean Association of Cardiopulmonary Resuscitation and the American Heart Association. Ethical approval was obtained from the institutional review board of Hallym University (HDT 2020-06-023).

### Selection of participants

To implement DL, it is essential to use a smart device, such as a tablet or videoconference system. To minimize the bias caused by inexperienced use of the device, high school students who are proficient in using smart devices were recruited. From July 1 to August 25, 2020, the Local Office of Education was asked to solicit participation from 28 high schools in the city. Students were considered for enrollment if they and their parents signed a letter of intent regarding participation and provided written consent for random assignment to the DL or conventional group. Subjects were excluded if they had physical or communication disabilities that would prevent them from performing CPR.

### Study protocol and intervention

Prior to the course, we checked whether subjects had previously received BLS training. It was found that some students had received only class BLS lectures without real practice. Therefore, in our study, we defined ‘previous BLS training’ as the case of completing the BLS training course in which the real practice is also included.

The conventional instructor-led training took place in a classroom setting and involved an instructional portion followed by skills practice. The instructional portion was transformed from a DVD format into a Kahoot! Format [11], while the practice-while-watching technique was maintained in its original format. The students practiced recognition of cardiac arrest, activation of the emergency response system, chest compressions, and automated external defibrillator (AED) use. Finally, the students performed hands-on practice of the entire process under the guidance of an instructor [12]. An Innosonian Brayden Pro^®^ manikin (Seoul, Republic of Korea) was used for visual feedback. The total training time was 60 min.

**Fig 1:**
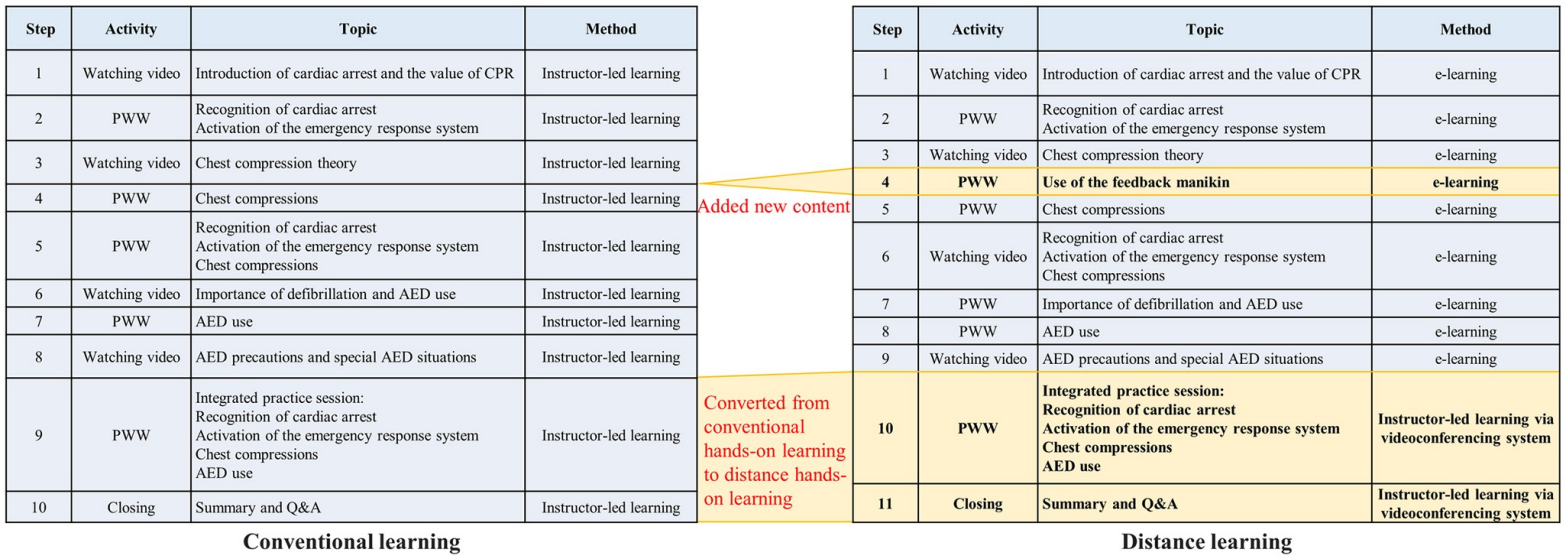
Training schemes for the conventional learning course and distance learning course. AED, automated external defibrillator; CPR, cardiopulmonary resuscitation; PWW, practice-while-watching.

**Fig 2:**
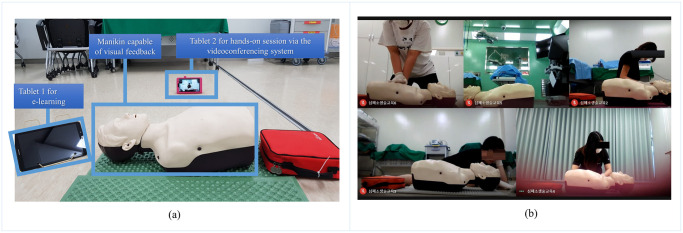
Environmental setting for the distance learning simulation (a) and videoconferencing system setting for the distance hands-on session (b).

The DL intervention was developed as a blended-learning format with an initial e-learning component followed by online real-time hands-on training using feedback devices under the guidance of an instructor. The e-learning component used the assignment function of Kahoot! with guidance regarding how to use the feedback manikin, as well as the same content from the conventional training. After finishing the e-learning component, including the assessments, the hands-on component was performed with an instructor via a videoconferencing system (Fig 1). Each isolated room at the training center contained two tablets, one CPR manikin, and an AED. One tablet was used to complete the e-learning component, and the other was used to monitor the student’s progress and facilitate communication between the instructor and student. The visual feedback manikin allowed the instructor to monitor the student’s CPR quality during the videoconference (Fig 2).

### Outcomes

The study team administered a questionnaire to the students to collect data regarding age, sex, and previous CPR training. To evaluate students’ performance before and after training, the instructor presented a situation where he found an adult who had collapsed on the road. After asking for help, the student was guided to perform the compression-only CPR alone for 2 minutes. Pre-training and post-training measurements of chest compression-related parameters were performed using Laerdal Resusci Anne QCPR^®^ manikins (Stavanger, Norway) without feedback. The parameters included compression rate, compression depth, proportion of accurate chest compressions, proportion of accurate chest compression depth, and complete release of compressions. Based on the current guidelines, the accurate chest compression rate was defined as 100–120 compressions/min, and the accurate chest compression depth was defined as 5–6 cm [13,14].

### Sample size

The sample size was calculated to detect an effect size of 20% with an α error of 5% and statistical power of 80% during two repeated measures conducted among the two groups. Based on these parameters, we estimated that a total sample size of 52 cases would be required. However, based on a presumed 20% exclusion rate, we aimed to recruit 33 subjects in each group and created 11 training sessions with six students per training session.

### Statistical analysis

Categorical variables were compared using the chi-squared test. Nonparametric continuous variables were analyzed using the Mann-Whitney U test. The pre-test and post-test comparisons in each group were performed using the Wilcoxon signed-rank test. As the normality assumption was not satisfied, non-parametric analysis of covariance (ANCOVA) was used to evaluate differences in chest compression quality before and after the different training methods. All statistical analyses were performed using SPSS software (version 25.0; IBM Corp., Armonk, NY, USA).

## Results

### Baseline characteristics

**Fig 3:**
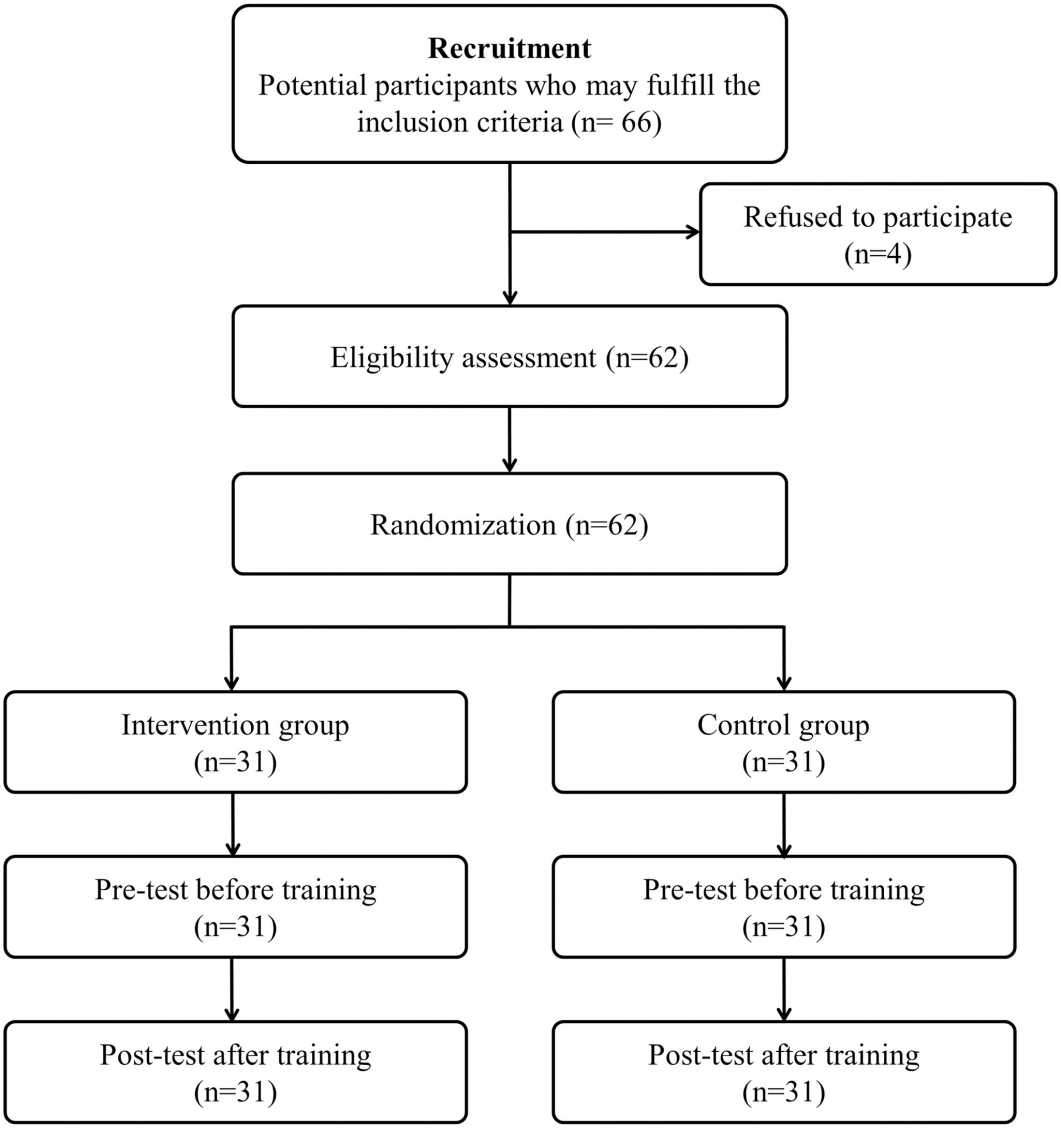
Flowchart for the cardiopulmonary resuscitation training and skill assessment.

A total of 62 participants were randomized to the conventional learning (CL) and DL groups (31 students in each group). All students completed the course without any dropouts (Fig 3). The median student age was 17 years (interquartile range [IQR]: 16–18 years), and 49 participants (79%) were female. There were no significant differences between the two groups in terms of age, sex, previous BLS training status, and timing of previous BLS training (Table 1).

**Table 1:** Baseline characteristics.

	CL (n = 31)	DL (n = 31)	p-value
Age, years	17 (16–18)	17 (16–18)	0.636
Sex, n (%)			0.211
Male	4 (12.9)	9 (29.0)	
Female	27 (81.1)	22 (71.0)	
Previous BLS course, n (%)	25 (80.7)	25 (80.7)	>0.999
Last BLS course, n (%)			0.153
Never	6 (19.4)	6 (19.4)	
<6 months earlier	11 (35.5)	9 (29.0)	
6–12 months earlier	13 (41.9)	10 (32.3)	
>12 months earlier	1 (3.2)	6 (19.4)	

Data are shown as median (interquartile range) or number (%). BLS, basic life support; CL, conventional learning; DL, distance learning.

### Effects of training

Table 2 shows a comparison of the participants before and after training. There was a significant change in the mean compression depth (before: 47 mm [IQR: 39–54 mm] vs. after: 49 mm [IQR: 45–54 mm], p<0.001). However, no significant changes were observed in the mean compression rate, proportion of correct compressions, proportion of correct compression depth, and proportion of correctly released compressions.

**Table 2:** Comparing the compression parameters before and after the training.

	Pre-training	Post-training	p-value
Mean compression rate, /min	116 (105.75–129.25)	117 (110.5–122.25)	0.481
Mean compression depth, mm	47 (39–54)	49 (45–54)	<0.001
Correct compression rate, %	34 (2.5–87.5)	67.5 (23.75–94)	0.066
Correct compression depth, %	35 (4.75–84.75)	48 (19–90)	0.061
Correct released compression, %	85 (45–97)	78 (28.75–98)	0.068

Data are shown as median (interquartile range).

### Main results

**Fig 4:**
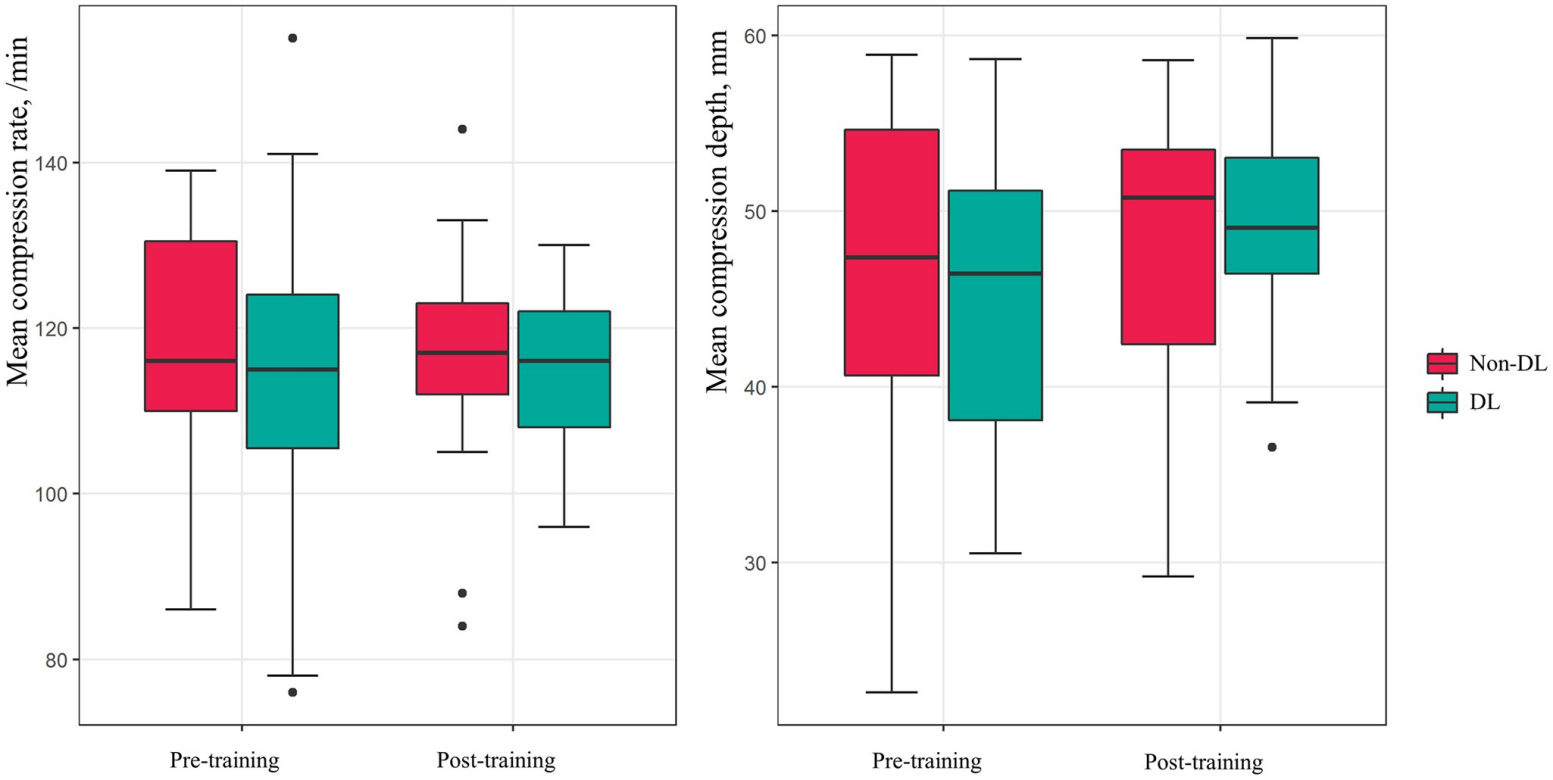
Pre- and post-training mean compression rates and mean compression depths from the non-DL and DL groups. DL, distance learning.

Table 3 shows a comparison of the CL and DL groups. There was no significant change in the mean compression rate and mean compression depth for the CL group before or after training. The DL group had a significant improvement in the mean compression depth (before: 46 mm [IQR: 37–52 mm] vs. after: 49 mm [IQR: 46–54 mm], p<0.001), although no change was observed in the mean compression rate (Fig 4). In addition, no significant changes were observed in the proportion of correct compressions, the proportion of correct compression depth, and the proportion of correctly released compressions. The ranked ANCOVA results revealed that the only significant difference between the CL and DL groups was observed in the mean compression depth (p = 0.015).

**Table 3:** Comparing the compression parameters before and after the training according to learning methods.

	CL (n = 31)			DL (n = 31)			Ranked ANCOVA	
	Pre	Post	p-value	Pre	Post	p-value	F	p-value
Mean compression rate, /min	116 (100–131)	117 (111–123)	0.638	115 (105–124)	116 (107–122)	0.511	0.343	0.560
Mean compression depth, mm	47 (41–55)	51 (42–54)	0.308	46 (37–52)	49 (46–54)	<0.001	6.334	0.015
Correct compression rate, %	34 (6–84)	42 (16–97)	0.198	55 (0–89)	71 (26–92)	0.084	0.018	0.894
Correct compression depth, %	43 (5–90)	60 (12–94)	0.785	34 (2–76)	47 (26–89)	0.258	0.241	0.625
Correct released compression, %	83 (45–97)	74 (32–94)	0.126	87 (45–98)	84(20–99)	0.045	2.204	0.143

Data are shown as median (interquartile range). ANCOVA, analysis of covariance; CL, conventional learning; DL, distance learning.

## Discussion

This study aimed to determine whether an online instructor-led DL course could improve CPR quality to the same extent as a CL course. The results revealed comparable improvements in CPR quality parameters between the DL and CL groups. Though it was only a slight difference, the DL group showed even more improvement in the mean compression depth compared to the CL group. To the best of our knowledge, this is the first study to evaluate the effectiveness of instructor-led DL using a videoconferencing system and a feedback device.

In the setting of cardiac arrest, BLS is considered the basis of resuscitation and one of the most important factors influencing survival, especially in cases of OHCA where a bystander can provide early CPR. In this setting, bystanders can immediately recognize cardiac arrest, activate the emergency medical system, and perform chest compressions [13]. Therefore, CPR training for laypersons is an important step in improving OHCA outcomes. The time and space constraints of face-to-face CL training have increased interest in online DL training, which has been complemented by recent technological innovations [10,15]. This study evaluated instructor-led online DL training in taking advantage of the benefits of instructor feedback and reduce the limitations of CL training.

Online DL can potentially reduce the required training time and resources and allow for standardization of education programs [16]. In addition, DL training has provided excellent results in terms of the initial assessment of trauma patients and is now recognized as a useful alternative for CL during the current COVID-19 pandemic [7,17]. Thus, there is increasing demand for DL BLS training, with one study identifying a 6-fold increase in enrollment and a 14-fold increase in course completion between April 2019 and April 2020 [18]. Other studies have indicated that video-based self-instruction is as effective as DL for CPR training and provided similar outcomes in terms of CPR skills (vs. CL training), with the exception of compression depth [9,19]. The suboptimal results for compression depth may be related to self-instruction without feedback, and the present study aimed to address this issue using instructor-led DL that permitted real-time feedback. The results from this strategy were generally equivalent to those of CL training, which suggests that instructor-led DL for CPR training can be as effective as CL training.

This study had several limitations. First, the sample size was small, although the number of participants permitted adequately powered the analyses. Second, we failed to assess the student’s retention of CPR knowledge. Third, the study involved high school students, and the results might not be generalizable to adults. Fourth, the prior BLS training experience might be associated with the students’ performance. Fifth, the compression depth changes before and after the training between the two groups was only 2 mm; therefore, it may lack clinical relevance. Thus, further studies are required to address these limitations.

In conclusion, instructor-led DL was suitable for CPR training and provided generally similar outcomes to those achieved via CL training. Thus, especially in settings where face-to-face CL is not practical (e.g., during the current COVID-19 pandemic), DL may be a useful tool for delivering CPR training.

## Supporting information

S1 Data (XLSX)
